# Genomic epidemiology of the UK outbreak of the emerging human fungal pathogen *Candida auris*

**DOI:** 10.1038/s41426-018-0045-x

**Published:** 2018-03-29

**Authors:** Johanna Rhodes, Alireza Abdolrasouli, Rhys A. Farrer, Christina A. Cuomo, David M. Aanensen, Darius Armstrong-James, Matthew C. Fisher, Silke Schelenz

**Affiliations:** 1 Department of Infectious Disease Epidemiology, Imperial College London, London, W2 1PG UK; 2 National Heart and Lung Institute, Imperial College London, London, SW3 6LR UK; 3 Department of Medical Microbiology, Charing Cross Hospital, Imperial College Healthcare NHS Trust, London, W6 8RF UK; 4 Broad Institute of MIT and Harvard, Cambridge, MA 02142 USA; 5 The Centre for Genomic Pathogen Surveillance, Wellcome Trust Genome Campus, Cambridgeshire, CB10 1SA UK

## Abstract

*Candida auris* was first described in 2009, and it has since caused nosocomial outbreaks, invasive infections, and fungaemia across at least 19 countries on five continents. An outbreak of *C. auris* occurred in a specialized cardiothoracic London hospital between April 2015 and November 2016, which to date has been the largest outbreak in the UK, involving a total of 72 patients. To understand the genetic epidemiology of *C. auris* infection both within this hospital and within a global context, we sequenced the outbreak isolate genomes using Oxford Nanopore Technologies and Illumina platforms to detect antifungal resistance alleles and reannotate the *C. auris* genome. Phylogenomic analysis placed the UK outbreak in the India/Pakistan clade, demonstrating an Asian origin; the outbreak showed similar genetic diversity to that of the entire clade, and limited local spatiotemporal clustering was observed. One isolate displayed resistance to both echinocandins and 5-flucytosine; the former was associated with a serine to tyrosine amino acid substitution in the gene *FKS1*, and the latter was associated with a phenylalanine to isoleucine substitution in the gene *FUR1*. These mutations add to a growing body of research on multiple antifungal drug targets in this organism. Multiple differential episodic selection of antifungal resistant genotypes has occurred within a genetically heterogenous population across this outbreak, creating a resilient pathogen and making it difficult to define local-scale patterns of transmission and implement outbreak control measures.

## Introduction

The emerging fungal pathogen *Candida auris* causes nosocomial invasive infections, predominantly in intensive care units (ICU). *C. auris* has a multidrug resistant (MDR) phenotype^1, 2^ with varying susceptibility to other azole drugs, amphotericin B and echinocandins^3–6^ and acquired resistance to fluconazole^7^.

Since its first description in 2009 in Japan^8^, *C. auris* infections have been reported in several countries^1, 4, 6, 9–16^ with identification routinely carried out by matrix-assisted laser desorption ionization time of flight mass spectrometry (MALDI-TOF MS)^11, 14, 15^. However, this method of identification can misidentify *C. auris* as other *Candida* species if commercial yeast identification databases are used^17^. Clonality has previously been identified within *C. auris* isolates from India, Brazil, South Africa, and South Korea using amplified fragment length polymorphism (AFLP) and multilocus sequence typing (MLST)^9, 14, 18^. However, given the low discriminatory power and reproducibility of these techniques, genetic relatedness between isolates cannot be investigated^14^; while not currently routine in the investigation of fungal outbreaks, whole-genome sequencing (WGS) provides increased power to assess the relatedness of isolates to analyze patterns of nosocomial infections and global spread^19^.

In 2016, we described the first large-scale *C. auris* outbreak (April 2015–November 2016) occurring within a single specialist cardiothoracic hospital in London^11^. Owing to the high uncertainty of the time and source of introduction of *C. auris* into the hospital, the rapid development of a molecular epidemiological toolkit was required. Outbreaks of other fungal pathogens have been previously investigated using short-read WGS, which provided sufficient information to discriminate between isolates and their phylogenetic relationships using single nucleotide polymorphism (SNP) analysis^20–22^. Recently, the handheld, portable MinION sequencer, manufactured by Oxford Nanopore Technologies, UK (ONT), has made rapid WGS widely available in the field and has been successfully used to analyze the molecular epidemiology of recent Ebola and Zika viruses outbreaks^23, 24^.

Here, we describe the use of MinION nanopore sequencing technology on a pathogenic fungus to determine the genetic epidemiology of this fungal outbreak, both within the UK hospital and within a global context, alongside a reannotation of the genome of *C. auris* and definition of novel antifungal resistance alleles.

## Results

We sequenced 25 clinical *C. auris* isolates from a recently described outbreak^11^, which included eight isolates derived from four patients taken several days apart to establish possible within-patient diversity. In addition, we also sequenced two samples from the hospital environment to better represent the overall genetic diversity within the impacted hospital.

### Rapid generation of outbreak *C. auris* reference genomes

We assembled five high-quality hybrid de novo reference genomes for *C. auris* using Illumina short-read sequences and MinION long-read sequences generated over 48 h. Five isolates were chosen to cover a range of dates (October 2015 to March 2016). Isolate 16B25 had the best overall assembly quality of 110 contigs, N_50 = _396,317 bp and a total sequence length of 12.3 Mb (Table 1; Supplementary Table S1). Approximately 98.94% of the 16B25 assembly mapped to the Pakistani *C. auris* genome B8441 assembled by Lockhart et al.^1^ Further analysis revealed that the unmapped 1.06% of the 16B25 assembly was distributed throughout the genome and was likely attributed to genetic variation between the two isolates.

**Table 1:** Summary of assembly and annotation statistics of the *C. auris* 16B25 genome, the *C. auris* B8441 reference^1^, the *C. auris* Ci 6684 reference^26^, and other pathogenic *Candida* species reference genomes^25^

Species	Genome size (Mb)	Number of chromosomes or scaffolds	GC content (%)	Number of protein coding genes	Average CDS size (bp)	Ploidy
*C. auris* (16B25)—hybrid assembly	12.3	110	45.13	5366	1564	Haploid
*C. auris* (B8441)	12.4	19	45.13	5439	1548	Haploid
*C. auris* (Ci 6684)	12.5	99	44.53	8358	1025	Haploid
*C. albicans* SC5314	14.3	7	33.5	6107	1468	Diploid
*C. tropicalis* MYA-3404	14.5	24	33.1	6163	1454	Diploid
*C. parapsilosis*	13.1	24	38.7	5733	1533	Diploid
*C. lusitaniae*	12.1	9	44.5	5941	1382	Haploid

We generated an average of 5.2 million Illumina reads passing quality control for 27 isolates recovered during the outbreak that mapped closely (average 95.5%) to our reference genome (Supplemental Material Table S2). A total of 5366 protein coding genes, 4 rRNAs, and 156 tRNAs were predicted using the genome annotation pipeline described in Supplementary Methods. Table 2 summarizes the general features of 16B25, along with those of other pathogenic *Candida* genomes. The number of protein coding genes presented here is in line with the predicted number of genes in *C. lusitaniae*^25^ (*n* = 5941), the closest known relative of *C. auris*.

There are fewer protein coding genes, tRNAs and rRNAs predicted in this genome than previously reported for *C. auris* Ci 6684 in Chatterjee et al.^26^, as shown in Table 1. Running our annotation pipeline on the B8441 isolate presented in Lockhart et al.^1^ found similar numbers of protein coding genes, rRNAs, and tRNAs (Table 1). Therefore, the different total numbers between 16B25 and Ci 6684 are likely due to different annotation pipelines and not the quality of the reference assemblies. The number of protein coding genes identified in Chatterjee et al. (*n* = 8358) was substantially greater than that found in other *Candida* species (Table 1) and is likely inflated due to over-prediction of short sequences, lack of filtering of repetitive sequences, and the use of only GenemarkS to predict the start of genes; our pipeline used additional criteria to achieve a predicted set of high-confidence genes.

### Phylogenetic analysis reveals an Indian/Pakistani origin of *C. auris* outbreak

**Fig. 1:**
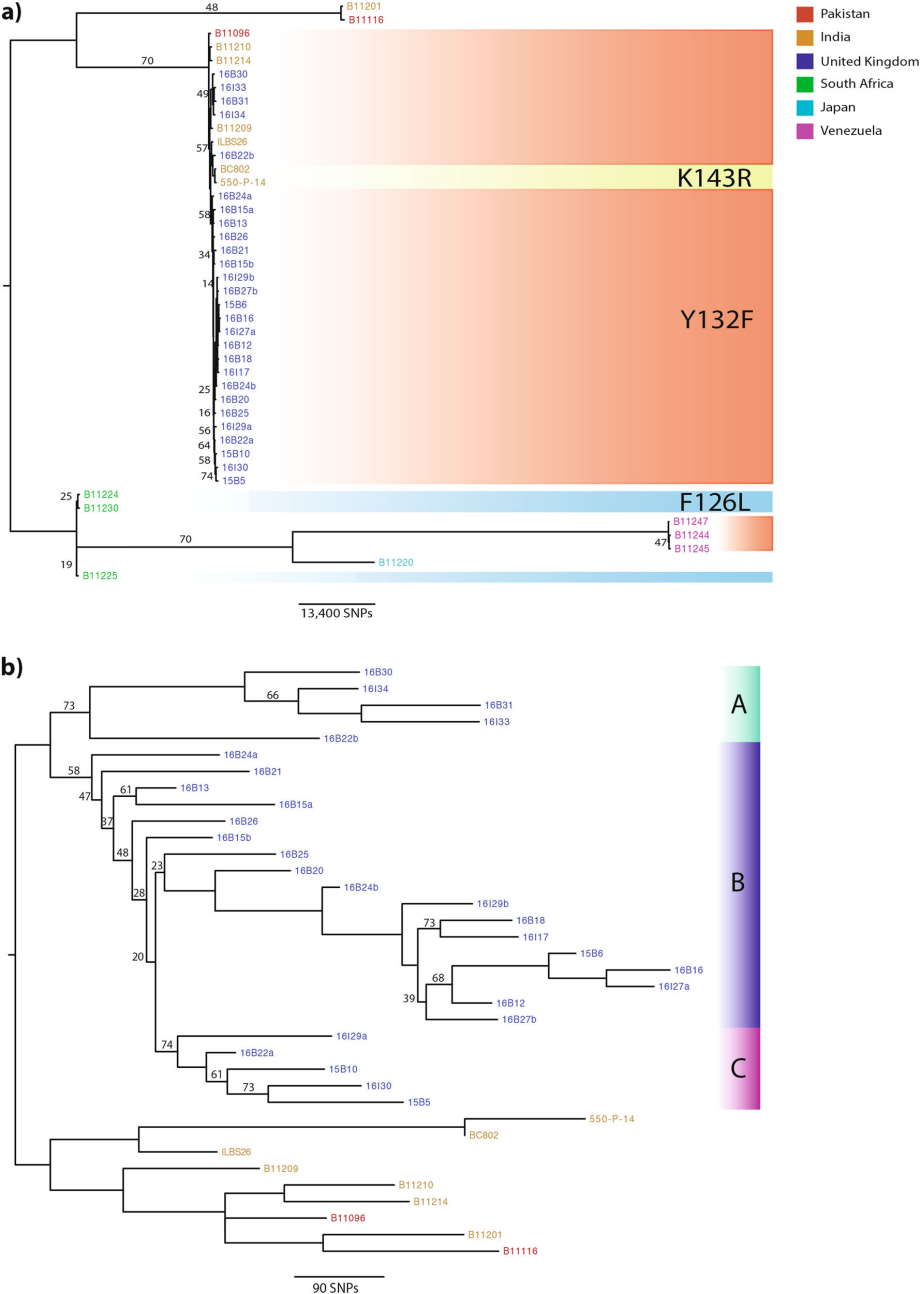
Phylogenetic analysis of *C. auris* isolates with bootstrap support (500 replicates) performed on WGS SNP data to generate maximum-likelihood phylogenies. Branches were supported 75% or higher unless otherwise stated. Branch lengths represent the average expected rate of substitutions per site. **a** Outbreak isolates from the UK (shown in blue) were combined with isolates from around the globe, including India (orange), Pakistan (red), Venezuela (pink), Japan (turquoise), and South Africa (green), to infer a possible geographical origin. Isolates with known mutations in the *ERG11* gene associated with resistance to fluconazole in *C. albicans* are shaded: Y132F in red, K143R in yellow, and F126L in blue. **b** Given the likely Indian/Pakistani origin of the outbreak isolates, phylogenetic analysis was repeated (as stated above), excluding isolates from South Africa, Venezuela, and Japan, to illustrate the UK outbreak. Isolates separating either into Cluster A (green), B (purple), or C (pink) are depicted to reflect likely introductions into the hospital

Phylogenetic analysis based on whole-genome SNPs revealed that the UK outbreak had an Indian/Pakistani origin (Fig. 1). SNP calls for isolates from Venezuela, India, Pakistan, Japan, and South Africa^1, 27^ were also included to add geographic context to the outbreak. The UK outbreak isolates were in the same clade as those from India and Pakistan (Fig. 1a); on average, 240 SNPs separated UK outbreak isolates from isolates collected in India and Pakistan. There were no known patient travel links to India or Pakistan prior to admission into the hospital, however. We found an average of 103 SNPs separating isolates within the UK outbreak; later isolates in Clade A towards the end of the outbreak (Fig. 1b) exhibited 134 SNPs separating them compared to earlier isolates in Clades B and C isolated at the start of the outbreak (Fig. 1b), which showed an average of 90 SNPs separating them.

**Fig. 2:**
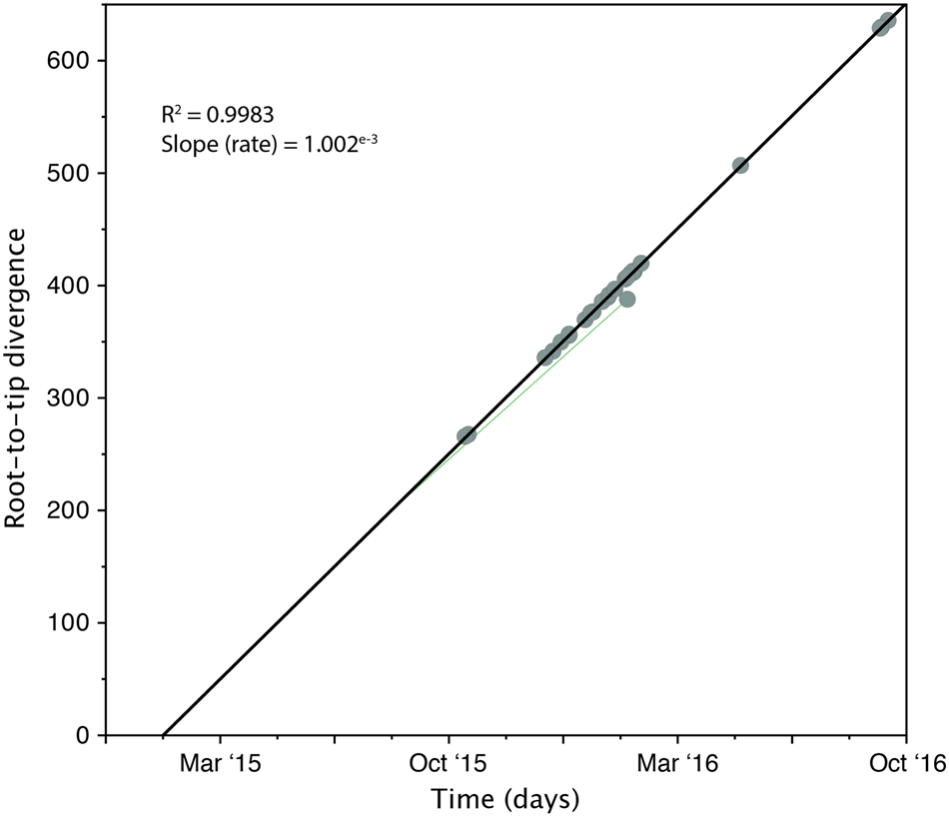
Root-to-tip regression analysis of all 27 *C. auris* outbreak isolates. Genetic distance is plotted against sampling time for the phylogeny of the *C. auris* outbreak. Each data point represents a tip on the phylogeny. The *R*^2^ for the regression and the slope, reflecting the evolutionary rate (in substitutions per site per day) is also shown

Fitting root-to-tip regression showed there was a linear relationship between sampling time (measured in days) and the expected number of nucleotide substitutions along the tree, demonstrating clock-like evolution across the timescale of the outbreak (Fig. 2). There was a strong association between genetic distances and sampling dates (*R*^2^* = *0.9992), showing that the data set has a reasonable temporal signal for further molecular clock analysis. The evolutionary rate of nuclear DNA (calculated from the slope of the regression) equated to 1.002e−3 substitutions per generation, which is comparable to nuclear DNA of other fungal species, such as *Schizosaccharomyces pombe* beer strains (3.0e−3^28^) and *Saccharomyces cerevisiae* (5.7e−3^29^). The time to the most recent common ancestor (TMRCA) was estimated to be late January 2015, two months prior to the first patient identified with a *C. auris* infection. One isolate, 16B15b, showed less genetic divergence from the root than expected given its date of sampling, which was perhaps an indication of excessive passage or recombination.

### Clinical isolates of *C. auris* show multidrug resistance

Out of the 27 *C. auris* outbreak isolates, six displayed reduced susceptibility to two or more classes of antifungal drugs, and thirteen isolates displayed reduced susceptibility to two or more azole antifungal drugs (Table 2). Only six isolates displayed reduced susceptibility to only one drug, fluconazole (Minimum Inhibitory Concentration (MIC): ≥256 µg/ml). Most (*n* = 24) isolates expressed elevated MICs to fluconazole (MIC: ≥256 µg/ml) with varying MICs to itraconazole (MIC: 0.03 µg/ml to ≥16 µg/ml), voriconazole (MIC: 0.12 µg/ml to 8 µg/ml) and posaconazole. Five isolates also displayed reduced susceptibility to amphotericin B (MIC: ≥2 µg/ml). Although resistance to amphotericin B is uncommon in *Candida* species^30^, studies have associated mutations in the *ERG2*, *ERG3*, *ERG5*, *ERG6*, and *ERG11* genes with the depletion of ergosterol and amphotericin B resistance^30, 31^. We did not identify any mutations in the aforementioned *ERG* genes in isolates displaying reduced susceptibility. Expanding the search across the genome found no candidate SNPs that were unique to isolates displaying reduced susceptibility.

**Table 2:** MICs at time of isolation of all isolates included in this study and associated molecular resistance mechanism identified in this study

Isolate ID	ANI	MICA	CAS	5FC	POS	VOR	ITR	FLU	AmpB	Molecular resistance mechanism
15B5	0.12	0.12	0.25	0.12	**8**	**8**	**16**	**256**	**2**	Y132F in *ERG11*
15B6	0.06	0.06	0.06	0.06	0.015	0.25	0.06	**256**	1	Y132F in *ERG11*
15B10	0.12	0.12	0.5	0.12	**8**	**8**	**16**	**256**	**2**	Y132F in *ERG11*
16B12	0.12	0.12	0.5	0.25	**8**	**8**	**16**	**256**	**2**	Y132F in *ERG11*
16B13	0.12	0.12	0.25	0.06	0.03	0.5	0.06	**256**	1	Y132F in *ERG11*
16I29b	N/A	N/A	N/A	N/A	N/A	N/A	N/A	N/A	N/A	Y132F in *ERG11*
16I29a	N/A	N/A	N/A	N/A	N/A	N/A	N/A	N/A	N/A	Y132F in *ERG11*
16B18	0.25	0.25	1	0.06	**0.12**	**2**	0.12	**256**	**1**	Y132F in *ERG11*
16B15a	0.12	0.06	0.12	0.06	0.008	0.06	0.015	16	0.5	Y132F in *ERG11*
16I17	N/A	N/A	N/A	N/A	N/A	N/A	N/A	N/A	N/A	Y132F in *ERG11*
16B20	0.06	0.12	0.5	0.06	**8**	**8**	**16**	**256**	1	Y132F in *ERG11*
16B16	0.12	0.12	0.25	0.06	0.03	0.25	0.03	**256**	1	Y132F in *ERG11*
16B21	0.12	0.12	0.25	0.06	**0.12**	0.5	0.03	16	1	Y132F in *ERG11*
16B22a	0.12	0.12	0.25	0.06	0.008	0.25	0.03	**128**	0.5	Y132F in *ERG11*
16B24a	0.12	0.12	0.5	0.06	**8**	**8**	**8**	**256**	2	Y132F in *ERG11*
16B22b	0.12	0.12	0.25	0.06	**4**	**8**	**2**	**256**	1	Y132F in *ERG11*
16B24b	0.12	0.12	0.25	0.06	**8**	**8**	**2**	**256**	1	Y132F in *ERG11*
16B25	0.12	0.12	0.25	0.06	0.03	0.12	0.03	**64**	1	Y132F in *ERG11*
16I27a	N/A	N/A	N/A	N/A	N/A	N/A	N/A	N/A	N/A	Y132F in *ERG11*
16B26	0.12	0.12	0.25	0.06	**8**	**8**	**16**	**256**	1	Y132F in *ERG11*
16B27b	0.12	0.12	1	0.12	**8**	**8**	**16**	**256**	0.5	Y132F in *ERG11*
16I30	0.25	0.12	0.5	0.12	**8**	**8**	**16**	**256**	**2**	Y132F in *ERG11*
16B15b	**8**	**8**	**8**	**64**	0.015	0.25	0.03	8	1	S652Y in *FKS1*; F211I in *FUR1*; Y132F in *ERG11*
16B30	0.12	0.06	0.12	0.06	**0.15**	0.5	0.06	**128**	1	Y132F in *ERG11*
16B31	0.12	0.06	0.12	0.06	0.03	0.5	0.06	**256**	1	Y132F in *ERG11*
16I33	0.06	0.06	0.06	0.06	0.06	0.06	0.03	**128**	0.5	Y132F in *ERG11*
16I34	0.06	0.06	0.06	0.06	0.008	0.25	0.03	**256**	0.5	Y132F in *ERG11*

MICs for FLU, ITR, VOR, POS, CAS, MICA AmpB, and ANI were above these tentative epidemiological cutoffs (ECOFF)^3^ and *C. albicans* ECOFF for 5FC^27, 60^ and are therefore resistant (in bold font)

*ANI* anidulafungin, *MICA* micafungin, *CAS*caspofungin, *5FC* 5-fluorocytosine, *POS* posaconazole, *VOR* voriconazole, *ITR* itraconazole, *FLU* fluconazole, *AmpB* amphotericin B, *N/A* no MICs carried out

One isolate (16B15b) displayed elevated MICs to all echinocandin drugs (MIC: ≥8 µg/ml) but remained susceptible to all azole drugs, with the exception to fluconazole. 16B15b also displayed reduced susceptibility to flucytosine (MIC: ≥64 µg/ml), which was not seen in the other isolates; therefore, resistance and any associated mutations were unique to this outbreak isolate. This isolate belonged to a patient who received anidulafungin for 7 days for pancolitis, developing *C. auris* candidemia 11 days afterwards, at which point isolate 16B15a was recovered. Treatment was switched to amphotericin and 5-flucytosine for 2 weeks. 6 days after completing this treatment, pan-resistant *C. auris* (16B15b) was recovered from the vascular tip. Previous studies in *Aspergillus* and *Candida* species identified mutations in *FKS1* associated with reduced echinocandin susceptibility or resistance^32, 33^; one non-synonymous SNP (nsSNP) causing a serine to tyrosine substitution (S652Y) was identified in the *C. auris FKS1* gene from the 16B25 reannotated genome. Our updated annotation protocol comparing multiple lines of evidence predicted an extended first exon for *FKS1* in comparison to the sequence submitted to GenBank (PIS58465); a comparison of the two protein sequences indicated that position 652 in the reannotated *FKS1* sequence is analogous to position 639 in the GenBank entry, resulting in S639Y in *FKS1* hotspot 1 (HS1). Another nsSNP caused a phenylalanine to isoleucine substitution (F211I) in the *FUR1* gene identified in the 16B25 reannotated genome, which has a role in 5-flucytosine resistance^34^. Although neither of these mutations has been previously reported, single nucleotide changes in *FUR1* in *C. albicans* and *C. lusitaniae* have been associated with 5-flucytosine resistance^34, 35^.

Orthologous sequences to *C. albicans ERG11* were screened for substitutions that conferred known fluconazole resistance mutations^36^. The Y132F substitution in *ERG11* was identified in all outbreak isolates, confirming an Indian/Pakistani origin. Lockhart et al.^1^ also found that these substitutions were strongly correlated with geographic clades.

### Interpretation of typing results in relation to epidemiology of the outbreak

*C. auris* outbreak isolates were grouped into three phylogenetic clusters (Clades A, B, and C; 18.5%, 63%, and 18.5%, respectively) (Fig. 1b). Clade A comprised five isolates from 2016 that had over 200 SNPs, distinguishing it from Clade B. On average, <100 SNPs separated the isolates in Clades B and C. Clade A was introduced into the hospital in early 2016 and formed the dominant outbreak strains towards the end of the outbreak; only 134 SNPs separated those isolates. Phenotypic antifungal susceptibility varied among Clade A isolates: all expressed elevated MICs to fluconazole (MIC: 128–256 µg/ml) and susceptibility to echinocandins (micafungin, caspofungin, and anidulafungin MIC: 0.06–0.12 µg/ml) and 5-flucytosine (MIC: 0.06 µg/ml) and two clinical isolates expressed reduced susceptibility to all azoles (MIC: 4–8 µg/ml posaconazole, 8 µg/ml voriconazole, 2–16 µg/ml itraconazole). This observation suggests that phenotypic antifungal susceptibility profiling cannot be reliably used to group genetically indistinguishable strains from nosocomial outbreaks.

Three patients with isolates in Clade A were admitted to the ICU. Two patients acquired *C. auris* while staying in the ICU, and another patient acquired *C. auris* within a surgical admission unit geographically placed next door to the high dependency unit in the same month, but this unit did not overlap with the ICU patients. Transmission of *C. auris* between different units within this hospital likely occurred via the movement of *C. auris*-positive patients or contaminated equipment. However, we are currently unable to establish routes of transmission in more detail because we did not sequence isolates from all patients during the outbreak and due to the heterogeneous nature of the founding population.

### Epidemiology of individual patient transmission during the outbreak

Eight isolates within this study were sequential pairs of isolates from four separate patients (Table 3); we hypothesized that there may have been nosocomial horizontal transmission between patients and/or their surrounding environment, as suggested in previous studies^4, 9, 10, 18^, for the following pairs of isolates: 16B22a and 16B22b from patient A (isolated 12 days apart); 16I27a (MICs were not carried out for this isolate) and 16B27b from patient B (isolated 1 day later); 16B24a and 16B24b from patient C (isolated 5 days apart); and 16B15a and 16B15b from patient D (isolated 32 days apart).

**Table 3:** Clinical isolates of *C. auris* included in this study

Isolate ID	Sequencing technology	Isolate date	Site	Origin
15B5	MinION, Illumina	19/10/2015	Vascath site	Patient
15B6	Illumina	22/10/2015	Swan ganz tip	Patient
15B10	Illumina	28/12/2015	Groin swab	Patient
16B12	Illumina	04/01/2016	Axilla	Patient
16B13	Illumina	11/01/2016	Femoral pulse index continuous cardiac output catheter site	Patient
16I29b	Illumina	18/01/2016	Bed	Environment
16I29a	Illumina	18/01/2016	Bed window trolley	Environment
16B18	Illumina	01/02/2016	Central venous pressure catheter tip in arm	Patient
16B15a	MinION, Illumina	06/02/2016	Blood culture	Patient D
16I17	Illumina	08/02/2016	Screen	Patient
16B20	MinION, Illumina	16/02/2016	Blood culture	Patient
16B16	Illumina	21/02/2016	Urine catheter	Patient
16B21	MinION, Illumina	22/02/2016	Axilla swab	Patient
16B22a	Illumina	27/02/2016	Axilla swab	Patient A
16B24a	Illumina	07/03/2016	Sputum	Patient C
16B22b	Illumina	09/03/2016	Femoral central venous pressure catheter	Patient A
16B15b	Illumina	09/03/2016	Central venous pressure tip	Patient D
16B24b	Illumina	12/03/2016	Pacing wire	Patient
16B25	MinION, Illumina	13/03/2016	Extracorporeal membrane oxygenation site	Patient C
16I27a	Illumina	14/03/2016	Screen	Patient B
16B26	Illumina	14/03/2016	Extracorporeal membrane oxygenation site	Patient
16B27b	Illumina	15/03/2016	Blood culture	Patient B
16I30	Illumina	21/03/2016	Screen	Patient
16I33	Illumina	16/06/2016	Groin	Patient
16B31	Illumina	16/10/2016	Central venous pressure line site	Patient
16B30	Illumina	17/10/2016	Groin	Patient
16I34	Illumina	23/10/2016	Axilla swab	Patient

In patient A, isolate 16B22a (recovered from the axilla) showed resistance to fluconazole only (Table 2). The subsequent isolate from this patient, 16B22b (isolated from a central line tip), exhibited resistance to all azole drugs (Table 2). Many SNPs (*n* = 277 SNPs) separated the two isolates (Fig. 1b) into distinct phylogenetic clusters (16B22a in Clade C and 16B22b in Clade A), suggesting independent acquisition of the infection by this patient within the unit. The two isolates from patient B similarly differed by many SNPs (*n* = 127 SNPs) but were both within Clade B. Given that 16I27a (from a body screen sample) was isolated 1 day prior to 16B27b (recovered from a positive blood culture), it is likely that this patient’s diversity represents an heterogenous *C. auris* population. In patient C, 16B24b was isolated from a clinical pacing wire sampled 5 days after the initial isolation of 16B24a from a sputum sample. These two isolates differ by 161 SNPs and were both placed in Clade B (Fig. 1b). In patient D, 16B15b showed raised MICs to all echinocandins and flucytosine, which were not seen in 16B15a. These two isolates were separated by 120 SNPs and were phylogenetically placed in Clade B (Fig. 1b). Our results suggest that none of these patients were infected with a single, clonally propagating *C. auris* strain; instead, the hospital (and all of its patients) was seeded with a genetically heterogenous population.

Three isolates (16B25, 16B20, and 16I29a) clustered closely together with an average difference of 99 SNPs. 16I29a was recovered from the environment around a *C. auris*-positive patient (the isolate was not sequenced) and was placed in Clade C (Fig. 1b). The patient from which 16B20 was recovered was present in an adjacent side room at the same time, suggesting a potential transmission between these patients, with 16I29a forming a new transmission chain of Clade C (Fig. 1b). 16B25 and 16B20 remained phylogenetically classified as Clade B (Fig. 1b). However, it remains unclear how the organism may have been transferred between the two rooms and patients. The third isolate of this cluster (16B25) was recovered from a patient present in the same room where 16I29a recovered. The patient was placed in this room 22 days after the previous *C. auris*-positive patient had left the room and became positive within 14 days of being in this isolation room.

Two isolates (16I29a and 16I29b) were isolated from the bed and trolley of a patient with a confirmed *C. auris* infection, who later died. However, MICs for these two environmental isolates were not carried out at the time of isolation. The genome sequence of the *C. auris* isolate from this patient was also not included in this study. A total of 161 SNPs differed between 16I29a and 16I29b, which were phylogenetically placed in Clades C and B, respectively. 16I29b appeared to be ancestral to a number of isolates in Clade B (Fig. 1b; 16B18, 16I17, 15B6, 16B16, 16I27a, 16B12, and 16B27b), which were, on average, separated by 122 SNPs.

## Discussion

*C. auris* is an MDR fungal pathogen with intrinsically reduced susceptibility to antifungal drugs^37^ that is capable of causing invasive infections. Here, we report WGS of *C. auris* infections from the largest UK outbreak to date, which occurred between April 2015 and November 2016 in a London hospital ICU and spread to two other wards.

A gold standard reference genome for the outbreak was assembled using long MinION-generated reads and Illumina short reads. While the GC nucleotide bias and base quality in >80% GC regions of the genome were similar in both Illumina and MinION sequencing, Illumina sequencing was more consistent across the whole genome (Figure S1). MinION reads displayed wide variation in base quality in >85% AT regions, ranging from a quality score of zero to 1.4, whereas Illumina reads ranged between quality scores of 0.75 and 0.85 for regions >85% AT. ONT has since released new chemistry that improves read quality and therefore variant calling, which may provide a competitive alternative to Illumina sequencing to further resolve transmission networks in outbreak settings and routine research.

Rapid generation of a genome assembly using MinION compliments the mapping of Illumina short reads to call high-confidence SNPs, which is of great importance in an outbreak^23, 24^. Indeed, the use of Illumina data alone would not have yielded an assembly of similar quality (Table S1), highlighting the need for long reads, such as those generated by MinION. SNPs in *ERG11* correlated with known *C. albicans* hotspots^36^, conferring resistance to the frontline drug fluconazole. Currently, no antifungal clinical breakpoints have been reported for *C. auris*^38^. All *C. auris* isolates in this study that exhibited high MICs to fluconazole (≥64 µg/ml) contained the Y132F amino acid substitution within *ERG11*, which is known to cause reduced susceptibility to fluconazole^36, 37^; seven isolates in which MIC determination was not carried out or the MIC was below the ECOFF of 64 µg/ml also contained amino acid substitutions known to cause reduced susceptibility to fluconazole. The ECOFFs at the time of publication are tentative^3^ and are not breakpoint MICs. Without performing the necessary allele swaps via gene deletion and observing wild-type phenotypes with lowered MICs to fluconazole, these *ERG11* mutations cannot be confirmed as the sole mechanism of reduced susceptibility in *C. auris*. Thirteen isolates with reduced susceptibility to fluconazole also displayed elevated MICs for voriconazole and/or itraconazole. This cross-:“resistance” to multiple azole drugs has been reported in other studies^4, 12, 39^, yet no mechanism has been reported. Expression level changes of *CDR1* have been associated with elevated itraconazole and fluconazole MICs in *C. glabrata*^40^, whereas *MDR1*, *MRR1*, and *GRP2* are differentially expressed in fluconazole and voriconazole resistant *C. albicans* isolates^41^. Differential expression of *ERG11*, *ERG6*, and *UPC2* was also associated with posaconazole resistance in *C. albicans*^41^. We did not identify a SNP-based mechanism responsible for the reduced posaconazole resistance in this study. Future studies therefore need to focus on the molecular biology of *C. auris* to elucidate the mechanisms of cross-resistance.

Five isolates displayed reduced susceptibility to amphotericin B, which although a rare event in *Candida* species, has been identified in up to 35% of *C. auris* isolates, suggesting an intrinsic resistance to this drug^1^. No SNPs were associated with this reduced susceptibility, suggesting that a non-mutation-based mechanism of resistance is responsible. Thirteen isolates were MDR for more than one azole drug, and six displayed reduced susceptibility to two or more classes of antifungal drugs, posing an important clinical challenge in the treatment of these *C. auris* infections. Within this study, we have also highlighted reduced susceptibility to posaconazole in addition to reduced susceptibility to echinocandin and flucytosine in one MDR isolate.

Echinocandin resistance is linked to mutations in the *FKS* genes in other *Candida* species^42^, and our analysis identified the S652Y mutation in *FKS1* in an echinocandin-resistant isolate; because the annotation pipeline in this study predicted an extended first exon, this mutation is analogous to position 639 in the *FKS1* sequence submitted to GenBank, resulting in a S639Y mutation in HS1. Previous studies have reported S639P and S639F mutations in *C. auris FKS1*^37, 43^; this finding suggests that different mechanisms of echinocandin resistance are present within the *C. auris* population, which is similar to previous observations in *ERG11* mutations^1^. Flucytosine resistance was observed in the same isolate and was associated with the F211I mutation in *FUR1*. Our analysis suggests that systemic echinocandin and flucytosine treatment can rapidly select for resistant genotypes across the outbreak timescale. Rapid MinION sequencing of *C. auris* isolates holds the promise of allowing the identification of drug resistance mutations, thus providing time-sensitive information in a clinical setting.

Phylogenomic analysis showed weak support for the monophyletic status of isolates within the hospital, suggesting that multiple introductions of *C. auris* occurred. On average, only 240 SNPs separated the UK outbreak isolates from the Indian/Pakistani clade, clearly showing that the UK outbreak had an Asian origin. When compared to other sequenced Asian *C. auris* isolates (Table S3) in Lockhart et al.^1^, these SNP numbers suggest an anomalous amount of diversity within this outbreak. Future alignments will require clade-specific references due to the large evolutionary distances between the South American/African and Indian/Pakistani clades. Although the mode of introduction into the UK is unknown, temporal analysis of the outbreak isolates placed the most recent common ancestor as early March 2015, which correlates closely to the first confirmed infection within the hospital one month afterwards, suggesting a recent introduction into the UK.

Only 161 SNPs separated hospital environment isolates (bed and trolley of a confirmed *C. auris*-infected patient). *C. auris* was also recovered from inanimate surfaces, suggesting a population of genotypes are capable of contaminating the hospital environment, causing onward infection of human hosts^1^. Given the SNP differences between isolates recovered from the same patient at different bodily locations (patient A, B, C, and D), the outbreak diversity is either due to multiple introductions, which is unlikely as all patients screened negative for *C. auris* upon admission, or a genetically heterogenous population seeded the hospital. Further, given the substantial number of SNPs separating the two isolates that were recovered from patient B 24 h apart, it is clear that genetically disparate isolates can infect the same host at the same time. The genomic diversity of *C. auris* within this outbreak makes mapping local-scale transmission events difficult as genetic bottlenecks may result in rapid changes in allele frequencies within local spatiotemporal scales. Clearly, sequencing multiple isolates of *C. auris* from patients alongside those from other UK outbreak settings is needed to more finely resolve the population genomic structure of this pathogen to better understand regional and local-scale transmission dynamics.

This study represents the first use of an ONT MinION sequencer on a human fungal pathogen; the rapid availability of long reads demonstrates that this technology is ideally used in an outbreak setting to provide high-quality contiguous assemblies. The association of a nsSNP in *FUR1* with flucytosine resistance is both clinically relevant and novel. Although previous studies have reported *FKS1* mutations, the S652Y mutation associated with echinocandin resistance presented here has not been previously reported. Further investigation into these mutations is required to confirm these associations.

Epidemiological analysis suggests that contact tracing was not sufficient for resolving fine-scale spatiotemporal processes across the outbreak due to multiple differential episodic selection events occurring across a genetically heterogenous *C. auris* population. Although the genomic approaches underpinning this study will likely be cornerstones of future research into this increasingly important pathogen, future research into *C. auris* should focus on sequencing many isolates from the same patient in multiple body sites to correctly establish the nature of persistence and transmission of *C. auris* within hospital environments. By combining molecular epidemiology with the traditional “shoe leather” epidemiology of contact tracing and interviewing patients, we will further our understanding of *C. auris* to achieve better control of future outbreaks.

## Methods

### Hospital and patient information

*C. auris* isolates were recovered from patients treated at the Royal Brompton Hospital (London, UK), a specialist cardiothoracic tertiary hospital. The outbreak was confined to adult patients in the mixed surgical and medical ICU, high dependency units (HDU) and the surgical admission ward (on a different floor). Details of the *C. auris* outbreak are reported in Schelenz et al.^11^ Echinocandin treatment was prescribed in suspected *C. auris* infections. If a urinary tract infection was suspected, combination therapy of amphotericin B and 5-flucytosine was prescribed. Clinical data were collected as part of an approved Royal Brompton Hospital Trust service evaluation.

### Fungal isolates

Twenty-seven *C. auris* isolates were studied, consisting of 25 clinical isolates from 21 patients and two isolates collected from the room of a patient known to be colonized with *C. auris* in the ICU (Table 3). The identification of *C. auris* was conducted by matrix-assisted laser desorption/ionization time of flight (MALDI-TOF) mass spectrometry (Bruker Daltonics, Fremont, CA, USA). An ethanol-formic acid extraction procedure was followed according to the manufacturer’s protocol for the identification of *C. auris* isolates. The spectra were analyzed using Flex Control 3.1 software (Bruker Daltonics Inc., Billerica, MA, USA) and MALDI Biotyper OC version 3.1 (Bruker Daltonics, Bremen, Germany). Scores were interpreted as >2.0 for species-level identification.

### Storage of *C. auris* isolates

All *Candida* isolates were stored in 50% glycerol solution at −70 °C. Isolates were subcultured onto Sabouraud dextrose agar (SDA; Oxoid Ltd., Basingstoke, UK) and incubated at 35 °C (±2°C) in ambient air for 18−48 h to ensure adequate growth when required for further experimentation.

### Antifungal susceptibility testing (AFST)

AFST of 9 systemic antifungal agents (amphotericin B, flucytosine, fluconazole, itraconazole, voriconazole, posaconazole, caspofungin, anidulafungin, and micafungin) was performed by colorimetric Sensititre YeastOne YST-10 broth dilution panels (Thermo Fisher Scientific, UK) according to the manufacturer’s instructions. Quality control was performed by testing *Candida krusei* ATCC® 6258 and *Candida parapsilosis* ATCC® 22019. All MICs were confirmed in the National Mycology Reference Laboratory, Public Health England, Bristol, UK, using broth microdilution according to CLSI method M27-A3^44^.

### gDNA extraction for MinION sequencing

*C. auris* isolates were grown in 10 ml of YPD broth (Sigma-Aldrich, UK) for 48 h at 37°C with agitation (180 rpm). High-molecular weight genomic DNA was extracted using the standard MasterPure Yeast DNA purification kit (Epicentre Biotechnologies, Cambridge, UK). Extracted gDNA was quantified using a Qubit 2.0 fluorometer and dsDNA BR (double-stranded DNA, broad range) assay kit (Life Technologies, Carlsbad, CA, USA). Quality control of gDNA prior to library preparation was performed using gDNA ScreenTape assays on the TapeStation 2200 system (Agilent, Santa Clara, CA, USA). Purified gDNAs were stored at 4°C until library preparation.

High-molecular-weight DNA (1 µg) was fragmented using a Covaris G-Tube (Covaris, Woburn, USA) at 4200 rpm. Fragmented DNA was end-repaired using the NEBNext Ultra II (New England Biolabs, Ipswich, USA), and cleaned using Agencourt AMPure XP beads (Beckman Coulter, USA) with a ratio of 1:1 beads to DNA mixture. End-repaired DNA was then A-tailed using the NEB Blunt/TA-ligase master mix (New England Biolabs, Ipswich, USA). The sequence-ready library was purified using MyOne Streptavidin C1 beads (Thermo Fisher Scientific, USA) and diluted prior to loading into the MinION flowcell. A 48-hour sequencing protocol was initiated using the MinION control software MinKNOW (v0.51.1.62). Read event data were base-called by the software Metrichor (v2.39.3). Each library was run on two flow cells to improve overall coverage.

### gDNA extraction for Illumina sequencing

High-molecular weight genomic DNA was extracted with an optimized MasterPure Yeast DNA purification kit (Epicentre Biotechnologies, Cambridge, UK) with an additional bead-beating step. Extracted gDNA was quantified as mentioned previously. Purified gDNA was stored at 4 °C until library preparation. All Nextera library preparation and Illumina HiSeq 2500 sequencing of 2 × 250 bp paired end reads was carried out by MicrobesNG (University of Birmingham, UK). Four additional isolates (16B30, 16B31, 16I33, and 16I34) from a later stage of the outbreak (from June 2016 to October 2016) were sequenced using TruSeq Nano library preparation and Illumina HiSeq 2500 sequencing of 2 × 250 bp paired end reads at The Centre for Genomic Pathogen Surveillance (Wellcome Genome Campus, Cambridge, UK).

### Library preparation and sequencing

Five clinical isolates (Table 3) representing a 146-day time frame were chosen for sequencing using the handheld MinION sequencer (Oxford Nanopore Technologies, Oxford, UK) to generate whole genome references and provide genome annotations to observe the merit of the sequencing technology.

Twenty-three isolates (Table 3), which include the five clinical isolates sequenced using MinION, were chosen for the current gold standard (Illumina sequencing) using the Nextera library preparation method as part of the MicrobesNG service (University of Birmingham, UK). The remaining four isolates were sequenced using TruSeq library preparation. These isolates represented a 155-day time frame and included isolates from patients and the environment around infected patients. Isolates of *C. auris* for both MinION and Illumina sequencing were cultured as described in supplementary methods. All raw reads in this study have been submitted to the European Nucleotide Archive under project accession PRJEB20230. Details of genome assembly and annotation are described in supplementary methods.

### Assembly of MinION and Illumina reads

MinION reads were extracted using poRe^45^. Hybrid assembly of Illumina and nanopore reads was performed for each isolate using SPAdes (v3.9.0) with default k-mer lengths (21, 33, and 55) and 1D scaffolding. An assembly statistics summary for each hybrid assembly is provided in Table S1. The hybrid assembly of isolate 16B25 provided the best statistics, so it was used as a reference for SNP calling. The genome assembly has been submitted to NCBI under the project accession PRJNA392455.

### Genome annotation

The *C. auris* genome was annotated using Genemark^46^, BLASTx against SwissProt^47^ and KEGG^48^, and HMMER hmmscan^49^ against PFAM^50^ and TIGRFAM^51^. We ran tRNAscan^52^ and RNAmmer^53^ to identify non-protein coding genes. Gene predictions were checked for a variety of issues, including overlap with non-coding genes, overlap with coding genes, and the presence of in-frame stops. Genes were named according to BLAST and HMMER evidence in the following order of precedence: (1) SwissProt, (2) TIGRfam, and (3) KEGG, where BLAST hits must meet the 70% identity and 70% overlap criteria to be considered a good hit and for the name to be applied. Otherwise, genes were named hypothetical protein. The gene annotations can be found at https://figshare.com/articles/Candida_auris_gene_annotations/5203618.

### Alignment of Illumina reads and phylogenetic analysis

Raw Illumina reads were quality checked using FastQC (v0.11.3; Babraham Institute) and aligned to the hybrid 16B25 reference genome using Burrows-Wheeler Aligner (BWA) v0.7.8 mem^54^ and converted to sorted BAM format using SAMtools v1.3.1^55^. Picard v2.6.0 and GATK v3.6^56^ were used to pre-process the alignments prior to variant calling. Base recalibration was also performed using GATK. Variants were called using GATK HaplotypeCaller, excluding repetitive regions as identified using RepeatMasker v4.0.6^57^, and filtered using parameters “DP < 10||MQ < 40.0||QD < 2.0||FS > 60.0||ABhom < 0.9”. Next, all variant calls that had a less than a minimum genotype quality of 50 were removed.

Phylogenies for whole genome SNP data were constructed and visualized as described in Rhodes et al.^58^ The rate of evolution (represented as the number of substitutions per day) along the tree topology was estimated using TempEst v1.5.1^59^ and calibrated with sampling times. Root-to-tip regression was calculated, and the root of the tree was selected to maximize *R*^2^.

### Benchmarking against CDC bioinformatics pipeline

Our bioinformatics pipeline was also used to call SNPs from isolates within the Indian/Pakistani clade presented in Lockhart et al.^1^ to confirm the genetic diversity between UK *C. auris* isolates in the outbreak; the results are presented in Table S3.

### Mutation identification in *ERG11*, *FKS1*, and *FUR1* genes

Orthologous sequences to *C. albicans ERG11* (SC5314) were extracted from each *C. auris* genome. Sequences were evaluated for amino acid substitutions to mutations within hot spot regions in *C. albicans*^36^ as described in Lockhart et al.^1^ Predicted *FKS1* and *FUR1* genes from the genome annotation were used to identify the presence or absence of mutations in *C. auris* isolates.

## Electronic supplementary material

Supplementary information
