# Supplementary material for: Genomic epidemiology of the UK outbreak of the emerging human fungal pathogen Candida auris
Source: Emerg Microbes Infect. 2018 Mar 29;7:43. doi: 10.1038/s41426-018-0045-x (PMC5874254; doi:10.1038/s41426-018-0045-x)
Supplement: Supplementary file 1 — Supplementary information [file 41426_2018_45_MOESM1_ESM.docx]

**Supplementary Information**

**Table S1: Details on input data and assembly statistics for each of the five isolates sequenced using MinION nanopore technology and Illumina sequencing.**

| Statistics | 15B5 | 16B21 | 16B25 | 16B25 – Illumina only | 16B20 | 16B15a |
| --- | --- | --- | --- | --- | --- | --- |
| Total Illumina paired-end sequences | 2910580 | 2418597 | 2467748 | 2467748 | 2616797 | 1888468 |
| MinION reads (bp) | 11213 | 10622 | 270395 | - | 5843 | 30415 |
| Average MinION read length (bp) | 2041 | 1689 | 1429 | - | 1628 | 2708 |
| Max MinION read length (bp) | 424741 | 317707 | 1231495 | - | 511629 | 834109 |
| No. contigs | 202 | 250 | 110 | 231 | 221 | 192 |
| Largest contig | 504599 | 418648 | 396317 | 278880 | 394313 | 329201 |
| Total length | 12382158 | 12390523 | 12322648 | 1231510 | 12378584 | 12369010 |
| GC (%) | 45.1 | 45.11 | 45.13 | 45.09 | 45.1 | 45. 11 |
| N50 | 124907 | 104174 | 176725 | 135650 | 123229 | 125352 |

**Table S2: Alignment statistics for Illumina reads mapped against the outbreak reference genome 16B25 for each outbreak isolate**

| Isolate ID | # QC-passed reads aligned (millions) | Depth of coverage (x) | % reference genome covered | # filtered high confidence SNPs |
| --- | --- | --- | --- | --- |
| 15B5 | 5.8 | 102 | 99.9 | 573 |
| 15B6 | 2.0 | 35 | 99.9 | 190 |
| 15B10 | 9.7 | 168 | 100 | 702 |
| 16B12 | 2.5 | 43 | 99.9 | 290 |
| 16B13 | 4.9 | 87 | 99.9 | 553 |
| 16I29b | 2.6 | 46 | 99.9 | 369 |
| 16I29a | 6.0 | 108 | 99.9 | 557 |
| 16B18 | 2.0 | 36 | 99.9 | 285 |
| 16B15a | 3.8 | 67 | 99.9 | 469 |
| 16I17 | 1.9 | 34 | 99.9 | 242 |
| 16B20 | 5.2 | 92 | 99.9 | 554 |
| 16B16 | 1.0 | 18 | 99.8 | 98 |
| 16B21 | 4.8 | 85 | 99.9 | 560 |
| 16B22a | 6.1 | 108 | 99.9 | 639 |
| 16B24a | 5.8 | 102 | 100 | 635 |
| 16B22b | 6.1 | 68 | 100 | 405 |
| 16B24b | 3.2 | 58 | 99.9 | 377 |
| 16B25 | 4.9 | 86 | 99.9 | 558 |
| 16I27a | 2.1 | 22 | 99.8 | 104 |
| 16B26 | 5.8 | 103 | 99.9 | 600 |
| 16B27b | 3.2 | 58 | 99.9 | 404 |
| 16I30 | 7.9 | 138 | 100 | 648 |
| 16B15b | 5.0 | 89 | 99.6 | 571 |
| 16B30 | 10.7 | 106 | 100 | 618 |
| 16B31 | 10.7 | 107 | 100 | 572 |
| 16I33 | 10.7 | 107 | 100 | 570 |
| 16I34 | 10.9 | 108 | 100 | 577 |

**Table S3: Comparison of the SNP calling pipeline presented in this study with the pipeline presented in Lockhart and colleagues** (1)**, with the average number of SNPs separating isolates stated in each study, suggests pipelines produce comparable results.**

| Geographical region | SNPs presented in Lockhart *et al.* | SNPs presented in Rhodes *et al.* |
| --- | --- | --- |
| South Asia | <60 | 53 |

**Figure S1**: Detailed metrics for reads that fall within windows of certain GC bins on our hybrid reference genome (constructed from long and short reads for isolate 16B25) for a) Illumina short reads only for isolate 16B25 and b) ONT long reads only for isolate 16B25.

**References**

1. Lockhart SR, Etienne KA, Vallabhaneni S, Farooqi J, Chowdhary A, Govender NP, et al. Simultaneous emergence of multidrug resistant Candida auris on three continents confirmed by whole genome sequencing and epidemiological analyses. Clin Infect Dis. Oxford University Press; 2017;64(2):134–40.
